# A Rare Case of an Aggressive Mixed Germ Cell Tumor Following Testicular Torsion

**DOI:** 10.7759/cureus.73935

**Published:** 2024-11-18

**Authors:** Joseph McGrath, Erick A Boldt, Shakil Huq, Dillin J Rhatigan, Matthew Lief

**Affiliations:** 1 Dr. Kiran C. Patel College of Osteopathic Medicine, Nova Southeastern University, Davie, USA; 2 Urology, Broward Health Coral Springs, Coral Springs, USA

**Keywords:** embryonal cell carcinoma, high afp, mixed germ cell tumor, radical orchiectomy, testicular malignancy, testicular mass, testicular torsion, urology and oncology, urology emergency

## Abstract

Testicular cancer is one of the leading malignancies affecting young men, with germ cell tumors (GCTs) being the most prevalent type. These tumors are classified into two main subtypes: seminomas and non-seminomatous germ cell tumors (NSGCTs), with the latter known for their higher likelihood of metastasis. Early detection through imaging and tumor markers like alpha-fetoprotein (AFP) and beta-human chorionic gonadotropin (HCG) is crucial for favorable outcomes. We present a case of a 40-year-old male patient with a recent history of a surgically repaired right testicular torsion presenting with progressive right testicular swelling and pain. Scrotal ultrasound revealed a markedly enlarged and heterogeneous right testicle measuring 5.4 cm, with mixed echogenicity and cystic features, highly suspicious for malignancy. Tumor markers were significantly elevated, with AFP at 19,420 ng/mL and HCG at 4,749 mIU/mL, indicating a high-risk testicular tumor. The patient underwent a right radical orchiectomy, which was complicated by tumor rupture during surgery. Pathology confirmed a testicular mixed GCT composed of embryonal carcinoma, yolk sac tumor, and teratoma with vascular/lymphatic invasion. This case highlights the importance of rapid intervention in managing NSGCTs, potential role of tumors in testicular torsions, and the unusual presentation of a rapidly growing NSGCT.

## Introduction

Testicular cancer, while relatively rare compared to other malignancies, is the most common cancer in young men aged 15-34 [1]. Among these cases, germ cell tumors (GCTs) make up over 95% of testicular neoplasms [2]. These tumors are divided into two main subtypes: seminomas and non-seminomatous germ cell tumors (NSGCTs). Seminomas are known for their slower growth and sensitivity to radiation, while NSGCTs, which include embryonal carcinoma, yolk sac tumor, teratoma, and choriocarcinoma, tend to exhibit more aggressive behavior and have a higher propensity for metastasis​. Despite its rarity, testicular cancer has an excellent prognosis, particularly when diagnosed and treated early​.

Several risk factors have been identified in the development of testicular cancer. The most significant of these include cryptorchidism (undescended testes), a prior history of testicular cancer, family history of testicular cancer, and certain genetic conditions such as Klinefelter syndrome [1]. Additional environmental and lifestyle factors, including exposure to endocrine-disrupting chemicals, have been suggested but remain under investigation [3]​. Interestingly, while testicular cancer is most commonly diagnosed in younger men, cases in patients over 40 are infrequent, and these cases tend to present with more advanced disease, underscoring the importance of early detection​. 

GCTs are highly responsive to treatment, but their aggressive potential, particularly among NSGCTs, necessitates rapid intervention. Seminomas tend to remain localized and exhibit a relatively indolent course, whereas NSGCTs are more prone to early spread via lymphatic and hematogenous routes. Mixed GCTs, containing both seminomatous and non-seminomatous elements, are often treated aggressively with both orchiectomy and adjuvant chemotherapy due to the risk of metastasis, even in cases where the seminomatous component is dominant [4]. Timely diagnosis, supported by imaging studies such as ultrasound and measurement of serum tumor markers, such as alpha-fetoprotein (AFP), beta-human chorionic gonadotropin (HCG), and lactate dehydrogenase (LDH), are critical in determining tumor type and staging.

We present the case of a 40-year-old male patient with a history of a right testicular torsion, which was managed with surgical detorsion and orchiopexy two months prior. The patient subsequently reported persistent pain and progressive swelling in the right testicle. Scrotal ultrasound revealed a heterogeneous mass measuring 3.5 cm in diameter with cystic features, highly suspicious for malignancy. Serum tumor markers were significantly elevated, with AFP and HCG levels suggesting the presence of an NSGCT. The patient underwent a radical orchiectomy, and subsequent histopathological examination confirmed a predominantly embryonal carcinoma mixed GCT containing yolk sac tumor, and teratoma.

This case highlights the unique challenges posed by testicular cancer in older patients, where presentation may be atypical and diagnosis delayed. Despite the low incidence of testicular cancer in this age group, clinicians must maintain a high index of suspicion when evaluating testicular masses in men over 40. Early detection, facilitated by thorough clinical evaluation and the use of imaging and tumor markers, remains the cornerstone of management, significantly improving prognosis and survival outcomes​.

## Case presentation

A 40-year-old male patient with a significant past medical history of hypertension and right testicular torsion presented to the office for an evaluation of right testicular pain and swelling for the past two weeks. The patient denied any urinary complaints, erectile dysfunction, hematuria and recent fever. The patient has a surgical history of an emergency right orchiopexy and right testicular detorsion two months prior to this visit for a testicular torsion. The patient reported that shortly after this procedure he noticed his right testicle progressively become more swollen and painful. 

The patient denied a family history of urological cancers as well as other cancers. The patient has three children with his wife and does not wish to have any more children. Further social history includes minimal alcohol use on social occasions, absence of smoking and illicit drug use history. The patient has a body mass index of 29.9. 

**Figure 1:**
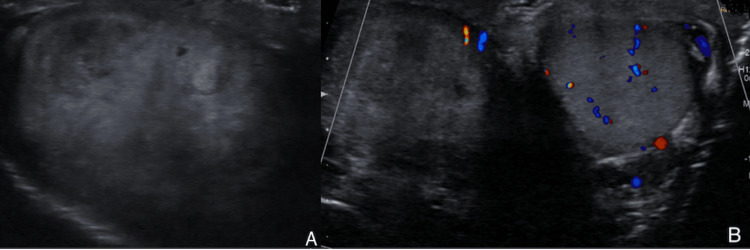
Scrotal ultrasound of an enlarged 5.4 cm diffusely heterogenous right testicle (A) and Doppler ultrasonography with minimal spectral Doppler flow indicating right testicular torsion (B)

Scrotal ultrasound with Doppler at the time of the testicular torsion incompletely evaluated the right testicle but noted a diffusely heterogeneous, enlarged right testicle measuring up to 5.4 cm with minimal color and spectral doppler flow compatible with a right testicular torsion and no masses (Figure 1).

**Figure 2:**
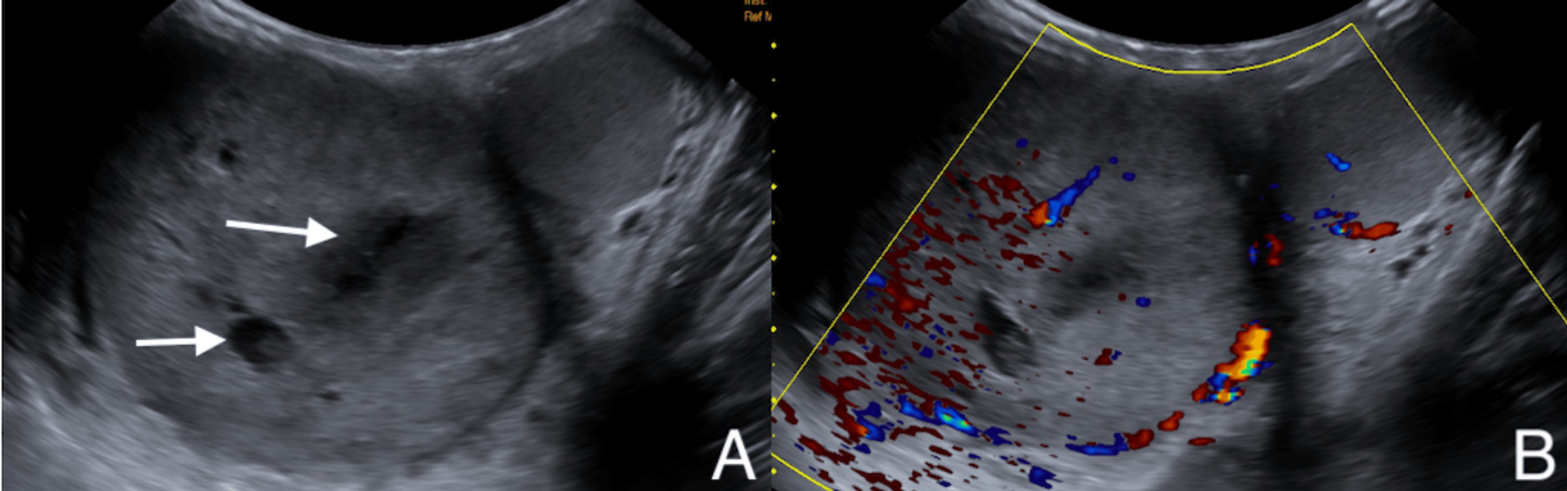
Scrotal ultrasound of an enlarged, heterogeneous right testicle with mixed echogenicity (A) and spectral doppler flow demonstrating increased vascularity (B)

Physical exam on initial encounter was significant for right testicle tenderness, edema, and a firm mass upon palpation. The left testicle was only mildly tender without any masses palpated. The patient was circumcised and has no urethral discharge. The patient had no signs of gynecomastia. Due to the suspicion of a testicular neoplasm, the patient was further evaluated with scrotal imaging and tumor markers. Scrotal ultrasound with Doppler revealed a markedly enlarged right testicle from prior sonogram that is heterogeneous with mixed hyperechoic and hypoechoic components and cystic features. There was no evidence of testicular torsion, hydrocele, or varicocele bilaterally on this sonogram. The overall impression was highly suspicious of right testicular malignancy and a significant enlargement of the right testicle compared to the prior scrotal sonogram (Figure 2).

The AFP tumor marker was significantly elevated at 19,420.0 ng/mL. Human chorionic gonadotropin (HCG) was significantly elevated at 4,749 mIU/mL. Prostate specific antigen (PSA) was within normal limits at 0.75 ng/mL. There is slight elevation in total testosterone of 840 ng/dL. Hepatic function panel results were within normal limits. The thyroid panel revealed a low T4 thyroxine level at 4.2 mcg/dL and low free T4 at 0.7 ng/dL with TSH and T3 within normal limits (Table 1). 

**Table 1:** Pertinent lab results before treatment

Parameter	Patient’s results	Reference range
Serum alpha-fetoprotein	19,420.0 ng/mL	<6.1 ng/mL
HCG, total	4,748 mIU/mL	<5 mIU/mL
Testosterone, total	840 ng/dL	250-827 ng/dL
PSA	0.75 ng/mL	≤4 ng/mL
T4 (Thyroxine), total	4.2 mcg/dL	4.9-10.5 mcg/dL
T4, free	0.7 ng/dL	0.8-1.8 ng/dL
TSH	0.57 mIU/L	0.40-4.50 mIU/L

The patient was promptly scheduled for a right radical orchiectomy as well as a left vasectomy at the patient’s request. The patient’s pre-procedural urine culture was negative for a urinary tract infection and subsequently received proper clearance for surgery. The patient was brought into the operation room and given general anesthesia. The left vasectomy was performed at the area of the external ring in order to not interfere with the scrotal anatomy.

Directly afterwards, a right radical orchiectomy was performed using a high inguinal approach. The ilioinguinal nerve was identified and kept away from the area of incision. Blunt dissection was utilized to separate the testicle from the dartos fascia. The right testicle was particularly fixed medially where it was suspected the prior testicular torsion was secured through the fascia. During the course of delivery, the right testicle through the inguinal canal, the tumor ruptured while inside the inguinal canal. The right testicle, right spermatic cord, and ruptured tumor were removed without contaminating the incision and collected for pathological evaluation. The wound was copiously irrigated with normal saline, and there were no signs of bleeding upon inspection. The scrotum was entirely intact, and there was no evidence of any ruptured tumor remains in the area of incision. The wound was then closed and the patient had no complications recovering from the procedure. 

The pathological evaluation of the right testicle confirmed a malignant mixed GCT consisting of embryonal carcinoma, yolk sac tumor (post-pubertal type), and teratoma (post-pubertal type). The tumor was predominantly embryonal carcinoma at 55% of the specimen. The size of the right testicle was 10.2 x 6.3 x 4.2 cm and weighed 80 grams. A cut section of the right spermatic cord revealed a firm encapsulated white tissue of 2.0 x 1.9 cm. The spermatic cord cut section revealed a firm encapsulated white tissue measuring 2.0 x 1.9 cm without tumor invasion. The ruptured tumor detached from the testis was a large brown oval shaped specimen measuring 10.2 x 7.8 x 5.3 cm and weighed 200 grams. The pathological stage of the mass was pT2 with the tumor being limited to the testis and epididymis with vascular/lymphatic invasion and no invasion through the visceral layer of the tunica vaginalis. Regional lymph nodes were unable to be assessed. 

**Figure 3:**
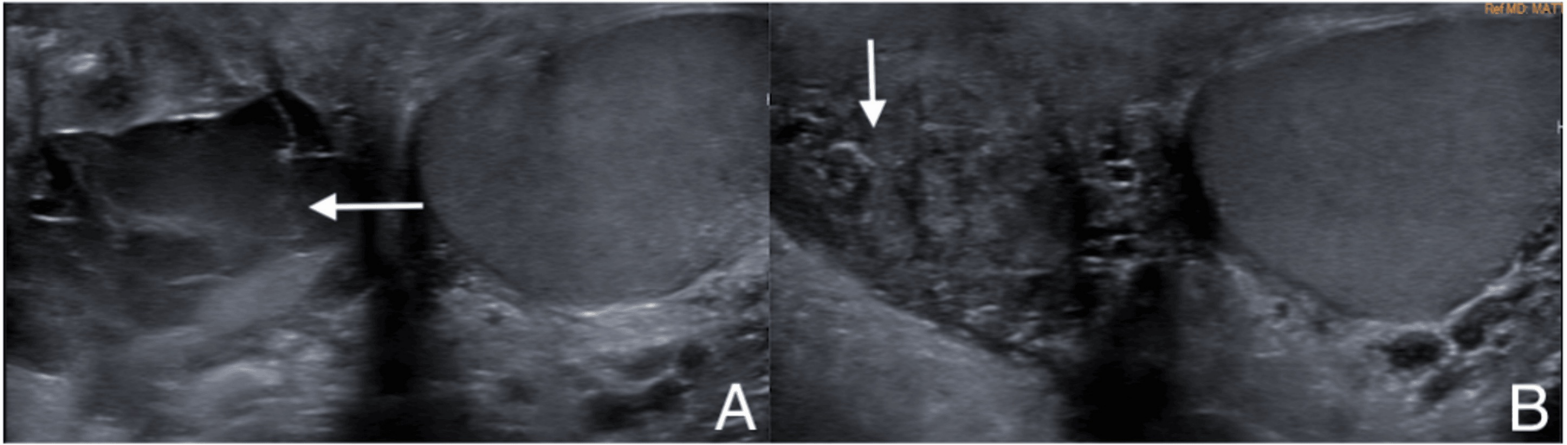
Post-right radical orchiectomy scrotal sonogram demonstrating complex fluid collection of 5.2 x 2.5 x 4.2 cm in right scrotal potential space

The patient developed a small hematoma on the right discovered on follow up examination 10 days post-operatively. The hematoma grew progressively and was evaluated with a scrotal ultrasound three weeks after the procedure which visualized complex fluid within the right scrotal potential space measuring 5.2 x 2.5 x 4.2 cm likely reflecting a hematoma (Figure 3). 

Post-orchiectomy CT of the chest, abdomen and pelvis with contrast had no findings to suggest metastatic disease. Due to the history of tumor rupture during the orchiectomy and aggressive nature of the tumor, the patient was referred to an oncologist and managed with four cycles of etoposide and cisplatin chemotherapy. The patient tolerated the chemotherapy regimen without complications and has had no signs of recurrence after completing the chemotherapy regimen.

## Discussion

This report underscores the uncommon association between testicular torsion and malignancy, the complexities of managing mixed GCTs, and the challenges that arise when a tumor ruptures during surgery. It also highlights the implications of elevated tumor markers on both prognosis and treatment strategies. In this context, we must examine the possible connection between testicular torsion and malignancy, the aggressive behavior of embryonal carcinoma, the risk of metastasis due to tumor rupture, and the importance of tailoring care to address both fertility and quality of life. Furthermore, the patient's significantly elevated tumor markers emphasize the need for aggressive treatment to minimize the risks of recurrence and metastasis. The following sections will explore these critical topics and highlight the importance of early intervention and close oncologic follow-up.

This case involves a 40-year-old male patient who presented with persistent right testicular pain and swelling two months after undergoing emergency orchiopexy and detorsion for testicular torsion. His post-operative course raised concern for malignancy, which was later confirmed to be a mixed GCT with embryonal carcinoma, predominantly.

Testicular GCTs represent the vast majority of testicular cancers, accounting for 90-95% of cases [5]. Mixed GCTs, like the one found in our patient, often contain multiple histological types, including embryonal carcinoma, yolk sac tumor, and teratoma. Embryonal carcinoma is known for its aggressive behavior, with a tendency for early vascular/lymphatic invasion, as seen in this case. The presence of vascular/lymphatic invasion, along with elevated tumor markers (AFP and HCG), and embryonal carcinoma predominance increased this patient's risk of metastasis and recurrence, even though post-orchiectomy imaging showed no evidence of metastatic disease [6].

The standard management of mixed GCTs with a benign contralateral testis starts with a radical orchiectomy with an inguinal approach, followed by adjuvant therapy based on pathological findings and staging [6]. In this case, the rupture of the tumor during the orchiectomy adds a layer of complexity. Tumor rupture within the inguinal canal increases the risk of local recurrence, necessitating close surveillance or potentially more aggressive adjuvant chemotherapy. Additionally, the pathological stage of pT2, indicating vascular/lymphatic invasion without spread beyond the tunica vaginalis, places the patient at higher risk for recurrence, further supporting the decision to refer him for chemotherapy.

The patient's prior history of testicular torsion could have been the initial presenting symptom of an underlying testicular tumor. The relationship between testicular torsion and malignancy is not well-established but has been observed in isolated case series. In one retrospective review of 32 patients with testicular torsion, 6.4% were found to have an underlying malignancy, suggesting a potential, albeit rare, association between torsion and testicular cancer [7]. While cryptorchidism is the most well-documented risk factor for testicular cancer, accounting for a 3.7-7.5-fold increase in risk, it is still important to evaluate for testicular masses potentially causing the torsion [6-8]. However, further studies are required to better understand this association and if testicular torsions affect the progression of malignancy through damaging the testicular parenchyma. 

The patient’s fertility and future reproductive options were carefully considered, given his personal history and desire not to have additional children. A left vasectomy was performed concurrently with his radical orchiectomy, consistent with the patient’s wishes. This decision highlights the importance of individualized care, taking into account both oncologic control and the patient’s quality of life post-operatively. It is important to note that adjuvant chemotherapy for NSGCTs has been shown to reduce fertility by 30%, so this adverse effect does not impact this patient since a vasectomy was performed during the procedure [6].

The significantly elevated tumor markers seen in this case, AFP at 19,420 ng/mL and HCG at 4,749 mIU/mL, place the patient in a high-risk category for NSGCTs. Tumor marker levels are crucial in determining the prognosis and treatment strategy for testicular cancer. AFP levels above 10,000 ng/mL and HCG levels over 5,000 mIU/mL are quite rare and considered high-risk tumors with a poor prognosis of 48% overall survival in 5 years [6]. Most patients with NSGCTs have lower marker levels, making this patient’s case unusual. For example, cases with AFP levels over 10,000 ng/mL are not common, occurring in about 5-10% of NSGCT patients, and HCG levels greater than 5,000 mIU/mL occur in around 8-10% of advanced cases​ [6,9].

These high levels are associated with an increased risk of tumor burden and aggressive disease, often requiring chemotherapy, as in this patient's case. The rupture of a testicular tumor during surgery is a serious complication, as it increases the risk of tumor cell seeding and local recurrence, potentially leading to metastatic spread [10,11]. Tumor rupture can expose adjacent tissues to malignant cells, which may spread beyond the confines of the scrotum and result in peritoneal or lymphatic dissemination. This is particularly concerning in NSGCTs due to their aggressive nature. Studies indicate that tumor spillage, especially with vascular or lymphatic invasion, can heighten the risk of local recurrence and distant metastasis [10]. Although the scrotum and inguinal canal were copiously irrigated in this case, the risk of microscopic cell seeding remains. A ruptured tumor has been associated with worsened outcomes in some cases, necessitating careful monitoring and often prompting earlier initiation of systemic chemotherapy.

Given the patient's elevated tumor markers, pathologic stage, and the presence of tumor rupture, current guidelines recommend close oncologic follow-up with chemotherapy for stage pT2 testicular cancer, even in the absence of detectable disease on imaging [12]. Chemotherapy protocols, including bleomycin, etoposide, and cisplatin (BEP), are standard for high-risk patients and have significantly improved survival outcomes in this population. Surveillance, while an option, may not be adequate in cases of vascular invasion and tumor rupture, further emphasizing the need for aggressive management [6,12]. Close post-operative surveillance, including imaging and serum marker tracking, is critical. Early referral to oncology for adjuvant chemotherapy is essential to address any potential micrometastatic disease and to prevent recurrence.

In summary, this case highlights the critical importance of aggressive management, early intervention, and careful follow-up in the treatment of mixed GCTs with complications such as tumor rupture and elevated tumor markers. The complexities of balancing oncologic control, fertility preservation, and quality of life further underscore the need for individualized patient care.

## Conclusions

This case underscores the complexity of testicular cancer presentation and management, especially in the context of a high AFP and HCG levels without evidence of metastatic disease. Despite aggressive behavior, early intervention, including radical orchiectomy and appropriate chemotherapy, offers favorable outcomes. The unique association between testicular torsion and testicular malignancy in this patient highlights the need for vigilant follow-up in similar cases. Further research into rapid AFP elevation and its clinical implications, particularly in non-metastatic settings, is essential to refine treatment protocols and improve patient prognosis.

## References

[1] Baird D, Meyers G, Hu J (2018). Testicular cancer: diagnosis and treatment. Am Fam Physician.

[2] Motzer RJ, Jonasch E, Agarwal N (2015). Testicular cancer, version 2.2015. J Natl Compr Canc Netw.

[3] Faja F, Esteves S, Pallotti F (2022). Environmental disruptors and testicular cancer. Endocrine.

[4] Arranz Arija JA, Del Muro XG, Caro RL (2024). SEOM-GG clinical guidelines for the management of germ-cell testicular cancer (2023). Clin Transl Oncol.

[5] Giona S (2022). The epidemiology of testicular cancer. Urologic Cancers.

[6] Nauman M, Leslie SW Nonseminomatous testicular tumors. StatPearls [Internet].

[7] Uguz S, Yilmaz S, Guragac A, Topuz B, Aydur E (2016). Association of torsion with testicular cancer: a retrospective study. Clin Genitourin Cancer.

[8] Ferguson L, Agoulnik AI (2013). Testicular cancer and cryptorchidism. Front Endocrinol (Lausanne).

[9] Marshall C, Enzerra M, Rahnemai-Azar AA, Ramaiya NH (2019). Serum tumor markers and testicular germ cell tumors: a primer for radiologists. Abdom Radiol (NY).

[10] Akpala A, Bhattacharyya S, Damola A, Viney R (2023). A rare case of ipsilateral scrotal recurrence of testicular cancer after radical orchidectomy. Cureus.

[11] Anna A, Saloni B, Adebiyi D, Viney R (2008). Recurrence of seminoma in the scrotum after orchidectomy. J Rural Med.

[12] (2024). EAU guidelines on testicular cancer. https://d56bochluxqnz.cloudfront.net/documents/pocket-guidelines/EAU-Pocket-on-Testicular-Cancer-2023.pdf.

